# Longitudinal strain analysis allows the identification of subclinical deterioration of right ventricular function in patients with cancer therapy-related left ventricular dysfunction

**DOI:** 10.15190/d.2019.7

**Published:** 2019-06-27

**Authors:** Diana Alexandra Cherata, Ionuț Donoiu, Rodica Diaconu, Adina Glodeanu, Doina Cârstea, Constantin Militaru, Octavian Istrătoaie

**Affiliations:** Department of Cardiology, “Filantropia” Municipal Hospital, Craiova, Romania; Department of Cardiology, University of Medicine and Pharmacy, Craiova, Romania; Department of Medical Semiology, University of Medicine and Pharmacy, Craiova, Romania

**Keywords:** Cardiotoxicity, right ventricle, cancer treatment related cardiac dysfunction, chemotherapy, right ventricle free wall longitudinal strain.

## Abstract

Background: This study was designed to assess right ventricular systolic function in cancer patients. Methods and Results: 68 consecutive patients receiving potentially cardiotoxic agents were followed for 6 months in a single-center, observational, cohort-study. Left ventricle and free-wall right ventricular longitudinal strain were analyzed prior and after 6 months of treatment, using a vendor-independent software, together with left ventricle ejection fraction, tricuspid annulus plane systolic excursion and right ventricular fractional area change. Cancer therapy-related cardiac dysfunction was defined as a left ventricle ejection fraction drop of >10% to <53%. Both left ventricle ejection fraction (59±7% vs. 55±8%, p<0.0001) and left ventricle longitudinal strain (−19.7±2.5% vs. −17.1±2.6%, p<0.0001) were reduced at follow up, along with free-wall right ventricular longitudinal strain (−24.9±4.5% vs. −21.6±4.9%, p<0.0001). Cancer therapy-related cardiac dysfunction was detected in 20 patients (29%). In 15 out of these 20 patients (75%), a concomitant relative reduction in free-wall right ventricular longitudinal strain magnitude by 17±7% was detected. Moreover, there was a significant correlation between left ventricle and free-wall right ventricular longitudinal strain at follow-up examinations (r=0.323, p<0.0001). A relative drop of right ventricular longitudinal strain >17% had a sensitivity of 55% and a specificity of 70% (AUC=0.75, 0.7-0.8, 95% CI) to identify patients with cancer treatment related cardiac dysfunction. Neither tricuspid annulus plane systolic excursion (24±5 vs. 23±4 mm, p=0.07), nor right ventricular fractional area change (45±8% vs. 44±7%, p=0.6) showed any significant change between examinations.Conclusions: Longitudinal strain analysis allows the identification of subclinical right ventricular dysfunction appearing in the course of cancer treatment when conventional indices of right ventricular dysfunction function are unaffected.

## **INTRODUCTION**

Cancer therapy-related cardiac dysfunction (CTRCD) diagnosis is conventionally based on the measurement of left ventricular ejection fraction (LVEF), using various cardiac imaging modalities. According to current recommendations, a LVEF decrease of more than 10% to a value of less than 53%, assessed by two-dimensional echocardiography (2DE), using the biplane Simpson’s method and demonstrated by repeated studies defines CTRCD^1^. Further characterization of CTRCD relies on presence/absence of symptoms and reversibility.

Induced cardiotoxicity expressed through CTRCD is most likely a continuous phenomenon characterized by progressive decline in LVEF. Because LVEF decreases when a critical amount of myocardial damage has occurred and cardiac compensatory mechanisms are exhausted, many affected patients may initially be asymptomatic, with clinical manifestations appearing even years later, often in the context of other triggering factors^2^.

Thus, detection of subclinical cardiac abnormalities, which may influence clinical decisions regarding the choice of chemotherapy, indication for cardioprotection or increased surveillance frequency (e.g. asymptomatic LV dysfunction) is crucial for cancer patients undergoing potential cardiotoxic regimes^3^. Furthermore, if CTRCD is detected early, patients frequently have a good functional recovery. Conversely, if patients are identified late after the onset of cardiac dysfunction, heart failure (HF) is typically more difficult to treat^4^.

Because LVEF measurement is a relatively insensitive tool for detecting early CTRCD, increasing efforts have been carried out in demonstrating the value of deformation imaging or strain for subclinical CTRCD detection. Myocardial deformation imaging performed using either Tissue Doppler Imaging (TDI) or speckle tracking echocardiography (STE) techniques allows the evaluation of LV and right ventricle (RV) myocardial mechanics.

LV global longitudinal strain (LVGLS) seems to be the most reproducible and useful deformation parameter in predicting subsequent CTRCD and HF before becoming clinically manifested^5^ and currently LVGLS represents a criterion for CTRCD diagnosis^2^: a relative reduction of more than 15% from baseline LVGLS suggests the risk of cardiotoxicity and further CTRCD.

On the other hand, RV function and mechanics have proven to be important indicators of overall cardiovascular morbidity and mortality^6-11^, and likewise, the clinical and prognostic value of RV strain analysis was demonstrated so far in patients with pulmonary hypertension, heart failure and LV assist devices, congenital heart diseases, storage diseases and cardiomyopathies with high risk of malignant ventricular arrhythmias^6-8^.

Although RV systolic function assessment through conventional parameters is recommended by the European and American expert consensus regarding cardiac function monitoring during cancer treatment^1,3^, RV free wall strain (RVFWLS) has been recommended as a more sensitive index of RV systolic dysfunction, being less load dependent and less influenced by heart motion than conventional parameters^11^.

However, the presence and the extent of RV function impairment during cancer therapy has not been adequately studied. Accordingly, this study was designed to assess RV systolic function in patients undergoing potential cardiotoxic treatments by longitudinal strain analysis and conventional parameters, using a vendor-independent, cardio-oncology dedicated software.

## MATERIALS AND METHODS

### ** *Study population*

Consecutive patients receiving potentially cardiotoxic agents and undergoing clinically indicated echocardiography studies for CTRCD monitoring, before and after 6 months of treatment, as main indication, were enrolled in asingle-center, observational cohort-study.

Patients were selected for acceptable image quality, excluding patients with two or more segments not visualized by conventional 2DE. Other exclusion criteria included severely dilated LV and LVEF less than 53% at baseline examination. For each patient the following data were obtained: patient demographics, cardiac risk factors and previous cardiac and malignancy history. All patients gave their informed consent in agreement with the local Ethics Committee.

### *Image acquisition*

Complete 2DE studies were performed prior and 6 months after receiving potential cardiotoxic treatment by experienced sonographers using commercially available ultrasound machines: Vivid E9 (GE Healthcare, Horten, Norway) and Philips IE33 (Philips Ultrasound) equipped with M5S probe and X5-1, respectively. In according to current guidelines^11^, four-, three-, and two- chambers apical LV views for LVGLS analysis and LVEF calculation were recorded, along with RV-focused apical four-chambers view for RV free wall longitudinal strain (RVFWLS) analysis and RV fractional area change (RVFAC) calculation. Additionally, tricuspid annulus plane systolic excursion (TAPSE) was measured using an M-mode cursor passed through the tricuspid lateral annulus.

All patients were examined in the left lateral position using grey-scale second-harmonic 2D imaging technique, with the adjustment of image contrast, frequency, depth, and sector size for adequate frame rate (50-80 fps) and optimal LV and RV border visualization. Care was taken to avoid LV and RV foreshortening, and image acquisition was done during breath-hold to minimize respiratory movements. Data sets were digitally stored in DICOM (Digital Imaging and Communications in Medicine) images-data format and exported to a separate workstation for offline analysis.

### *Image analysis*

**Figure 1:**
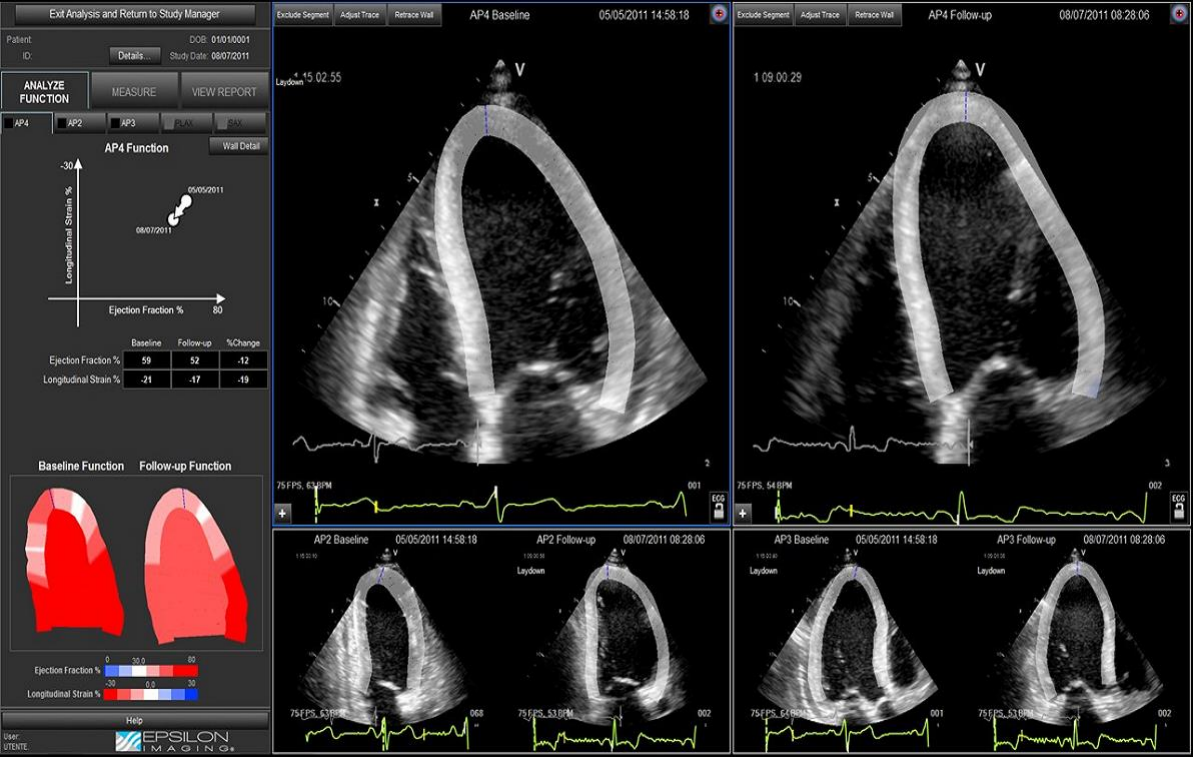
Example of simultaneous baseline and follow up two-dimensional speckle-tracking analysis of the left ventricle (LV) using a vendor independent software and using DICOM images. In this figure the region of interest (ROI) was tracked and left ventricular ejection fraction (LVEF) and LV global longitudinal strain (LVGLS) were computed: A. Baseline analysis of apical four chambers view; B. Follow-up analysis of apical four chambers view; C. Baseline analysis of apical two chambers view; D. Follow-up analysis of apical two chambers view; E. Baseline analysis of apical three chambers view; F. Follow-up analysis of apical three chambers view; G. Integrated display of changes in LVEF and LVGLS from baseline to follow-up; H. LVEF and LVGLS baseline and follow up values and changes as proportions of the initial value. I. Baseline and follow up colored map of the ventricle wall.

A semi-automated vendor-independent software (Echo-Insight Cardio-Oncology, Epsilon Imaging, Ann Arbor, MI) was used to load simultaneous baseline and follow up studies **(Figure 1)**. The method was applied independently in all patients and in random order, on digitally stored DICOM images-data format, by the same observer (D.A.C.), blinded to the clinical data.

Briefly, after the end-diastole and end-systole of the LV apical four-, three- and two-chambers views frames were identified using frame-by-frame analysis and based on mitral valve closure and electrocardiogram (ECG) trace, endocardial boundary was traced manually, using an interrupted mouse clicks technique and tracked throughout the cardiac cycle based on an automated STE algorithm, resulting in LV volumes and peak systolic LVGLS over time. LVGLS and LVEF were automatically calculated by the software for both baseline and follow up studies, as following: LVGLS as the mean peak systolic strain value measured in all 2D points in the three apical views (18-segments model) and LVEF as the corresponding end-systolic and end-diastolic volumes in four-and two- chambers LV views, using biplane disc-summation algorithm (modified Simpson’s rule).

**Figure 2:**
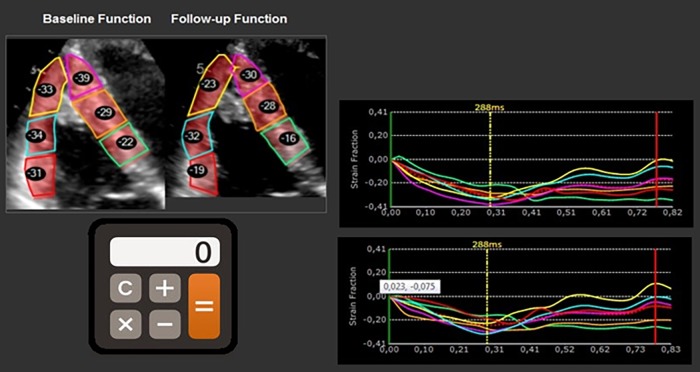
Successful tracking of a 6-segment region of interest (ROI) in a right ventricular (RV)-focused apical four-chamber view. The operator may either approve the tracing or manually exclude the septal segments by clicking on the respective segments. Then, RV free wall longitudinal strain (RVFWLS) was computed by averaging the peak systolic strain values of the three segments of the free wall (from base to apex), displayed by software.

RV analysis was performed for baseline and 6 months follow up studies for each patient, using the same automated STE algorithm as for LV. First, tracing of the entire RV endocardial border in RV-focused apical four-chamber view was performed, obtaining RV global longitudinal strain (RVGLS) (including the septum) strain value. Then, RVFWLS was computed by averaging the peak systolic strain values of the three segments of the free wall (from base to apex), displayed by the software **(Figure 2)**. This approach was preferred to RVGLS, because it is based on the inclusion of the interventricular septum that may partially reflect changes in the LV, as the septum is shared by both ventricles^11^.

RVFAC was automatically calculated after manually tracing RV endocardial border after end-diastole and end-systole frames were identified, paying attention to include the trabeculated endocardium within the RV cavity.

Using the tricuspid lateral annulus M mode RV focused four-chamber view, TAPSE was manually traced, as the distance between end-diastole and peak systole excursion of the RV annular plane towards the apex.

Finally, the software then generated an integrated display of changes in LVEF and LVGLS from baseline to follow-up **(Figure 2)**. Changes between baseline and follow-up studies in LVEF, LVGLS, RVFWLS, RVFAC and TAPSE – noted with the sign Δ, were automatically reported in proportions of the original value. E.g. a change in LVGLS value from −20% to −10% was reported as ΔLVGLS = 50%.

### *Statistical analysis*

Variables are expressed as mean ± standard deviation (SD). Contingency tables were applied to characterize the demographic and clinical profile of the study group. Pearson test was used to investigate the correlation power between variables. The normal distribution was measured univariate (one by one), using Kolmogorov Smirnov test. Homoscedasticity, as the multivariate normality, was checked with a M-Box test, for a p < 0.05.

We used stepwise Discriminant Analysis (DA) to set the predictive power of the variables for CTRCD. DA was preferred to Logistic Regression because it is a more solid and robust analysis for small samples size. Assumptions were made by differentiating patients in three groups: (1) LVEF drop with > 10% to < 53%, (2) RVFWLS > −20% (< 20% in magnitude with the negative sign) from group (1), and (3) patients with no CTRCD. The differences of variables in each group were investigated in first place, by the ANOVA test. A p value < 0.05 was considered statistically significant. Firstly, the DA found the most important classificatory factors (most predictive variables using ANOVA test). Secondly, the DA identified the discriminating functions that can explain the variability of the groups, discovering which one is more important in each function. Finally, DA checked the predictive power of the functions, and assigned the probability to predict individually enclosure to one or another group. The independent variables that were classified by DA are important predictors for CTRCD.

Receiver operating characteristic (ROC) analysis was used to display results about the sensibility and specificity of the studied variables to predict CTRCD.

Data were analyzed using SPSS version 22.0 (SPSS, Inc, Chicago, IL, USA).

## **RESULTS**

A total of 155 patients were addressed for an echocardiography study for CTRCD monitoring between October 2014 and August 2016 at the echocardiography laboratory of the Department of Cardiology, Emergency County Hospital, Craiova, Romania. Among these patients, 107 (69%) had at least one follow-up examination after 6 months and only 68 (43%) met our inclusion criteria. The remaining patients were either not sent for follow up examinations by the treating hematologist/oncologist, had an echocardiography study with poor image quality, had LVEF < 53% or were followed by other Cardiology centers.

68 patients from Hematology and Oncology departments were followed for 6 months. Demographics, clinical characteristics and malignancy history of the study patients’ group are described in **Table 1**. 90% of CTRCD group patients (n = 18) were treated with anthracycline-based scheme: doxorubicin and cyclophosphamide followed by either bleomycin or vincristine and monoclonal antibody-rituximab: R-CHOP, R-FCM, R-BEACOP schemes, while only 10% of patients (n = 2) were treated with bortezomib, melphalan and corticosteroids (VMP scheme).

**Table 1:** Demographics, clinical characteristics and malignancy history of the study patients *breast: 2, lung: 1, digestive cancers: 2, thymoma: 1

Variables	All patients (n = 68)	CTRCD patients (n = 20)
Males (%) Age (years) Body surface area (m2) Heart rate (bpm) Systolic blood pressure (mmHg) Diastolic blood pressure (mmHg)	51% (n=35) 55 ± 14 (range: 21-86) 1.84 ± 2.14 79 ± 13 127 ±17 75 ± 12	45% (n=9) 56 ± 15 years (range: 27-75) 1.79 ± 3.17 84 ± 10 120 ±16 84 ± 10
Cardiac risk factors and history: High blood pressure Diabetes Smoking Coronary Artery Disease Valvular heart disease	16% (n=11) 4,4% (n=3) 13% (n=9) 7,3% (n=5) 5,6% (n=4)	20% (n=4) 5% (n=1) 10% (n=2) 5% (n=1) 20% (n=4)
Malignancy history: Hodgkin’s Lymphoma Non-Hodgkin Lymphoma Multiple Myeloma Acute myeloid leukemia Acute lymphoblastic leukemia Other Tumors*	13,2% (n=9) 29,4% (n=20) 25% (n=17) 14,7% (n=10) 10,3% (n=7) 7,4% (n=5)	25% (n=5) 55% (n=11) 15% (n=3) 5 % (n=1) - -

All study patients (n = 68) had normal pre-chemotherapy LVEF values.

Both LVEF (59 ± 7% versus 55 ± 8%, p < 0.0001) and LVGLS (−19.7 ± 2.5% versus −17.1 ± 2.6%, p < 0.0001) were reduced at follow up. RVFWLS also decreased in magnitude (−24.9 ± 4.5 versus −21.6 ± 4.9, p < 0.0001). Neither TAPSE (24 ± 5 versus 23 ± 4 mm, p = 0.07), nor RVFAC (45 ± 7 versus 44 ± 8%, p = 0.6) showed any significant change between baseline and follow-up examinations** (Table 2)**.

**Table 2:** Echocardiographic parameters before and after treatment

Echocardiographic parameters	Initially	After 6 months	P value
LVEF (%)	59 ± 7	55 ± 8	p < 0.0001
LVGLS (%)	−19.7 ± 2.5	−17.1 ± 2.6	p < 0.0001
RVFWLS (%)	−24.9 ± 4.5	−21.6 ± 4.9	p < 0.0001
TAPSE (mm)	24 ± 5	23 ± 4	p = 0.07

Taking into consideration the cut-off value for CTRCD definition^1^, a study sub-group resulted: patients with LVEF drop of > 10% to a value of < 53% (29%, n = 20) or CTRCD patients’ group **(Table 1)**.

LVEF was significantly reduced to 46 ± 7.5% in CTRCD patients’ group, compared to pre-treatment mean values of 58 ± 8% (p < 0.0001). LVGLS was abnormal during CTRCD, decreasing to −14 ± 2% versus −18 ± 2% (p < 0.0001), being significantly lower than the published lower limit of normal value for LVGLS of > −18 % (< 18 with the negative sign)^7^.

Changes between baseline and follow-up studies: ΔLVEF, ΔLVGLS, ΔRVFWLS, ΔRVFAC and ΔTAPSE, were automatically reported in proportions of the original value.

ΔLVGLS decreased by 20 ± 7%, being even higher than the reported abnormal limit of 15% change from baseline to follow-up LVGLS, significant enough to predict subclinical CTRCD^1,2^.

In the most up-to-date version of the cardiac chamber quantification guidelines for echocardiography^11^, an abnormality threshold for the RVFWLS was set at > −20% (< 20% in magnitude with the negative sign)^11^, resulting that 15 out of 20 patients experiencing CTRCD (75%) in our study had RVFWLS abnormal values. Interestingly, among these patients, RV dysfunction evaluated by conventional parameters was present in only 5 patients by RVFAC, while by TAPSE reduction in only 3 patients.

RVFWLS was significantly decreased compared to baseline (−17 ± 2% versus −24.6 ± 2%, p = 0.005) during CTRCD and ΔRVFWLS was 17 ± 7%. RVFAC was significantly lower at follow-up for CTRCD patients (44 ± 9% versus 41 ± 8%, p = 0.06), although still in the normal range (considering an abnormal RVFAC of < 35%)^11^. The same scenario was noted for TAPSE (24 ± 4 mm baseline versus 21 ± 3 mm, p = 0.05) for < 17 mm as lower limit of normal value^11^ **(Table 3)**.

**Table 3:** Echocardiographic parameters in CTRCD patients’ group before and after treatment

Echocardiographic parameters	Pre-treatment	After treatment	P value
LVEF (%)	58 ± 8	46 ± 7.5	p < 0.0001
LVGLS (%)	−18 ± 2	−14 ± 2	p < 0.0001
RVFWLS (%)	−24.6 ± 2	17 ± 2	p = 0.005
RVFAC (%)	44 ± 9	41 ± 8	p = 0.06
TAPSE (mm)	24 ± 4	21 ± 3	p = 0.05

Moreover, significant correlation resulted between follow-up RVFWLS and LVGLS (r = 0.323, p < 0.0001), RVFWLS and LVEF (r = 0.279, p < 0.1) and RVFWLS and female gender (r = 0.279, p < 0.1).

Stepwise DA found that next to ΔLVEF, ΔRVFWLS is an important factor for predicting CTRCD, meaning that ΔLVEF by itself is not enough to explain the differences between groups. The probabilities of classification of those functions obtained with DA are approximately 97% for individual classifying.

**Figure 3:**
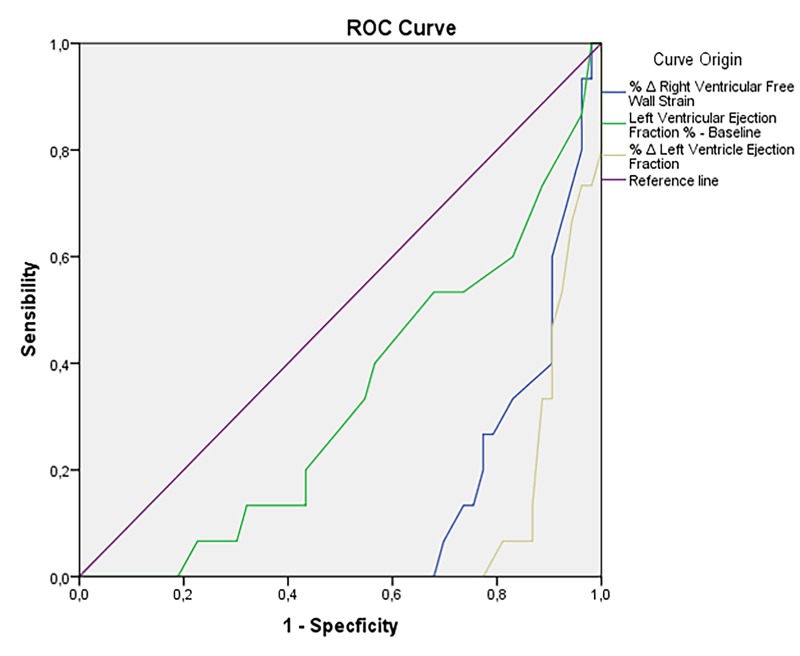
The receiver operating characteristic (ROC) curve analysis for cancer-therapeutics related cardiac dysfunction (CTRCD) patients group established that a relative drop of right ventricular (RV) free wall longitudinal strain (RVFWLS) > 17% had a sensitivity of 55% and a specificity of 70% (AUC=0.75, 0.7–0.8, 95% CI) to identify patients with CTRCD.

The ROC curve for CTRCD group **(Figure 3)** established that a relative drop of RVFWLS > 17% had a sensitivity of 55% and a specificity of 70% (AUC=0.75, 0.7-0.8, 95% Cl) to identify patients with CTRCD.

At the time of CTRCD, 2 patients (10%) reported symptoms consistent with class II-III New York Heart Association (NYHA) HF and had severely abnormal LVEF (< 30%), while the rest of the patients were classified as class I NYHA HF with mildly abnormal LVEF (41-53%).

## **DISCUSSION**

Our study demonstrates that in patients experiencing CTRCD, right ventricular function is lower than baseline as measured using RVFWLS. RV dysfunction was seen in 25% of CTRCD patients by RVFAC, a conventional parameter, analogous to LVEF, and in up to 75% patients based on the strain analysis by STE, using a vendor independent, cardio-oncology specific software. The proportion of patients with abnormal RVFWLS was larger than those with abnormal RVFAC, demonstrating the sensitivity of strain measures to identify subtle RV dysfunction.

Currently, RV dysfunction is not considered in the diagnosis of CTRCD and its incidence and prognostic value in patients receiving potential cardiotoxic therapy have not been adequately studied. The limited literature on the impact of cancer therapy on the RV may reflect the absence of robust techniques for the assessment of RV function. However, given the thinner structure of RV with fewer myofibrils, the RV may also be susceptible to damage by the cardiotoxic cancer therapy, as we have shown in this study. In fact, American Society of Echocardiography (ASE) and European Association of Cardiovascular Imaging (EACVI) experts’ consensus on proper cardiac care of adult patients receiving cancer treatment recommends RV function monitoring during cancer therapy.

The effect of cancer therapy on the RV was first demonstrated in an older study on 41 patients with various cancers and with anthracycline-based treatment by radionuclide ventriculography and based on wall motion abnormalities^12^. More recently, 46 women with breast cancer receiving anthracyclines were studied using cardiac magnetic resonance (CMR) and showed RV dysfunction in 34% of the patients after 12 months follow-up, while LV dysfunction was seen in 26%^13^. In the same study, RV dysfunction was present as early as after 4 months of therapy, and was interpreted as an early sign of myocardial injury. An echocardiography study identified mild reduction in RVFAC and TAPSE in 37 patients with breast cancer treated with anthracyclines^14^, although mostly in the normal range.

Two studies of 19 and 56 survivors of pediatric cancers and treated with anthracyclines have shown a reduction in RVFWLS as a marker of subclinical ventricular dysfunction^15,16^. Interestingly, in a more recent study of patients with advanced HF receiving LV mechanical circulatory support, patients with CTRCD were significantly more likely to also require RV mechanical support^17^.

The incidence of concomitant RV dysfunction at the time of CTRCD by RVFWLS has been recently studied in 30 patients with HER2+ breast cancer treated with trastuzumab with or without anthracyclines and concomitant RV dysfunction was seen in up to 40% of the patients^18^. The study showed a lower propensity for subsequent ventricular function recovery although this did not reach statistical significance due to the small sample size.

Investigations that have followed breast cancer patients undergoing anthracycline-based chemotherapy for at least one year agree that right ventricular structure, systolic/diastolic function and mechanics are significantly impaired^19,20^. Moreover, RVFWLS sensitively predicts dyspnea development in breast cancer patients receiving epirubicin therapy^21^.

Future work should be conducted to determine if right heart dysfunction precedes left heart abnormalities, potentially permitting earlier detection and possible intervention strategies to prevent chemotherapy-induced cardiac dysfunction in this particular population.

Our work builds on the existing literature demonstrating that in patients experiencing CTRCD, longitudinal strain analysis allows the identification of subclinical RV dysfunction when conventional indices of RV function are unaffected.

A recent study by Cardinale et al.^22^, involving 2625 patients (mean follow-up 5.2 years), showed a 9% overall incidence of cardiotoxicity after anthracycline treatment, and 98% of cases occurred within the first year and were asymptomatic. In our study cardiotoxicity incidence was 29%, including symptomatic and asymptomatic LVEF drop along with transitory CTRCD.

RVFWLS computed by the average from the three segments of the RV free wall was preferred to other techniques based on our clinical experience that noted this approach as being the most feasible and reproducible^23^.

Ideally, LVGLS should be considered alongside LVEF when interpreting longitudinal data for CTRCD monitoring, and moreover, including RV function assessment. Currently, strain measurements are dependent on the analysis software, which is usually limited to proprietary formats generated by a specific imaging system. The major limitation for the use of strain parameters is the inter-vendor variability, resulting in dependency on the analysis software, which is usually limited to proprietary formats generated by a specific imaging system.

The new Position Paper of the European Society of Cardiology (ESC) on cancer treatments and cardiovascular toxicity^3^ suggests that: early detection of high-risk patients, prompt diagnosis of CTRCD, the use of imaging modalities that provide additional relevant clinical information (e.g. RV function) and consistency regarding the imaging modality assay for continued screening throughout the treatment pathway, should be fundamental tasks for the management of cancer patients, involving both cardiologists and oncologists.

In this sense we used a vendor-independent with a dedicated cardio-oncology platform, which uses DICOM images for the analyses of volumes and strain, in an easy-to-understand display and rapid interpretation of serial images.

In a head-to-head comparison of LVGLS measurements using STE software packages from seven different ultrasound machine vendors and two software-only vendors, the performance of nonvendor-specific software did not differ from that of vendor-specific packages^24^. It was noted a moderate but statistically significant bias between vendors that was, however, within acceptable limits for most combinations of software packages. Authors concluded that LVGLS may be safely used in routine clinical practice but should preferably be interpreted relative to previous examinations with the same machine and software or versus vendor-specific reference values.

RVFWLS appears to be reproducible and feasible for clinical use, but because of the need for additional normative data from large studies involving multivendor equipment, no definite reference ranges are currently recommended for either global or regional RV strain or strain rate.

### *Limitations*

This was a retrospective study from a single center with a relatively small sample size. However, the low rates of CTRCD at our center and the lack of universally accepted follow-up explain our small sample size. Furthermore, we used an age and cardiac risk factor balanced control group of Hematology and Oncology patients who had echocardiography prior to any cancer treatment to account for this limitation.

After the end of chemotherapy, follow-up data was present only in 35% (n = 7) of the CTRCD patients. This reflects the retrospective nature of the study, and the fact that many patients are not routinely followed with cardiac imaging at our tertiary care center once cancer therapy is completed. Therefore, it is not possible to estimate ventricular function recovery; we consider this as the most important limitation. Further studies are needed to define the clinical and prognostic significance of RVFWLS reduction in cancer patients and whether LV dysfunction recovery would be influenced during follow-up in patients with RVFWLS decrease. Furthermore, concomitant RV dysfunction may have implications for cardiac therapy in patients experiencing cardiotoxicity.

In our study patients have a certain variability in the way their malignancy was treated. However, all patients received potential cardiotoxic therapy with a majority (75%) having received anthracyclines. We also did not report chemotherapy dose/kg as it was not the objective of our study.

Finally, we did not do a logistic regression because the sample size was too small to obtain meaningful results.

### *Clinical Implication*

The number of studies demonstrating that right ventricular structure, function and mechanics are valuable predictors of cardiovascular and total morbidity and mortality in patients with a wide range of cardiovascular conditions is constantly increasing.

The presence of RV dysfunction during cardiotoxicity in our study demonstrates the need to assess both ventricles function during cancer therapy. This is consistent with recent ESC guidelines, which encourages routine follow-up of both LV and RV functions during cancer treatment. Our finding is a hypothesis that needs to be confirmed in larger studies.

The appropriate length of cardioprotection or HF treatment in patients experiencing cardiotoxicity during cancer therapy is unknown, but definitely it is influenced by the coexistent RV dysfunction. In addition, these patients may need close cardiology follow-up.

RV dysfunction may be a marker of more significant cardiac injury and a potential risk factor for persistent LV dysfunction during follow-up.

Combined results and collective evidence generated using different echocardiographic parameters will provide deeper insights into the pathology of this intriguing pathology, ultimately translating into more accurate diagnosis and better clinical management of cardiovascular complications in cancer patients. Incorporating new echocardiographic parameters for LV and RV assessment into clinical cardio-oncology trials may ultimately prove useful in defining the cardiotoxic profile of new and existing chemotherapeutic agents.

## **CONCLUSION**

Longitudinal strain analysis allows the identification of subclinical RV dysfunction in patients with cancer therapy-related LV dysfunction when conventional indices of RV function are unaffected. This demonstrates the need to assess both ventricles function in patients receiving potential cardiotoxic treatment that may have major implications in cancer patients’ management.

Better prediction of CTRCD may support the identification of patients who can benefit by closer surveillance during and after exposure to potentially cardiotoxic chemotherapy, decrease the number of cardiac complications and further increase the life expectancy of patients suffering from cancer.

Further studies are needed to define the clinical and prognostic significance of RVFWLS reduction in cancer patients.

## **KEY POINTS**

**◊** **The presence of right ventricle dysfunction demonstrates the need to assess both ventricles function during cancer therapy**

**◊** **Right ventricle dysfunction is an early sign of myocardial injury in cancer therapy-related cardiotoxicity, and longitudinal strain analysis allows the identification of subclinical dysfunction when conventional indices of right ventricle function are unaffected**

**◊** **Future work should be conducted to better understand the place of the right ventricle in the spectrum of cancer therapeutics-related cardiac dysfunction**

## References

[1] Plana J. C., Galderisi M., Barac A., Ewer M. S., Ky B., Scherrer-Crosbie M., Ganame J., Sebag I. A., Agler D. A., Badano L. P., Banchs J., Cardinale D., Carver J., Cerqueira M., DeCara J. M., Edvardsen T., Flamm S. D., Force T., Griffin B. P., Jerusalem G., Liu J. E., Magalhaes A., Marwick T., Sanchez L. Y., Sicari R., Villarraga H. R., Lancellotti P. (2014). Expert consensus for multimodality imaging evaluation of adult patients during and after cancer therapy: a report from the American Society of Echocardiography and the European Association of Cardiovascular Imaging. European Heart Journal - Cardiovascular Imaging.

[2] Eschenhagen Thomas, Force Thomas, Ewer Michael S., de Keulenaer Gilles W., Suter Thomas M., Anker Stefan D., Avkiran Metin, de Azambuja Evandro, Balligand Jean-Luc, Brutsaert Dirk L., Condorelli Gianluigi, Hansen Arne, Heymans Stephane, Hill Joseph A., Hirsch Emilio, Hilfiker-Kleiner Denise, Janssens Stefan, de Jong Steven, Neubauer Gitte, Pieske Burkert, Ponikowski Piotr, Pirmohamed Munir, Rauchhaus Mathias, Sawyer Douglas, Sugden Peter H., Wojta Johann, Zannad Faiez, Shah Ajay M. (2011). Cardiovascular side effects of cancer therapies: a position statement from the Heart Failure Association of the European Society of Cardiology. European Journal of Heart Failure.

[3] Zamorano Jose Luis, Lancellotti Patrizio, Rodriguez Muñoz Daniel, Aboyans Victor, Asteggiano Riccardo, Galderisi Maurizio, Habib Gilbert, Lenihan Daniel J., Lip Gregory Y. H., Lyon Alexander R., Lopez Fernandez Teresa, Mohty Dania, Piepoli Massimo F., Tamargo Juan, Torbicki Adam, Suter Thomas M. (2016). 2016 ESC Position Paper on cancer treatments and cardiovascular toxicity developed under the auspices of the ESC Committee for Practice Guidelines. European Heart Journal.

[4] Cardinale Daniela, Colombo Alessandro, Lamantia Giuseppina, Colombo Nicola, Civelli Maurizio, De Giacomi Gaia, Rubino Mara, Veglia Fabrizio, Fiorentini Cesare, Cipolla Carlo M. (2010). Anthracycline-Induced Cardiomyopathy. Journal of the American College of Cardiology.

[5] Thavendiranathan Paaladinesh, Poulin Frédéric, Lim Ki-Dong, Plana Juan Carlos, Woo Anna, Marwick Thomas H. (2014). Use of Myocardial Strain Imaging by Echocardiography for the Early Detection of Cardiotoxicity in Patients During and After Cancer Chemotherapy. Journal of the American College of Cardiology.

[6] Hardegree Evan L., Sachdev Arun, Villarraga Hector R., Frantz Robert P., McGoon Michael D., Kushwaha Sudhir S., Hsiao Ju-Feng, McCully Robert B., Oh Jae K., Pellikka Patricia A., Kane Garvan C. (2013). Role of Serial Quantitative Assessment of Right Ventricular Function by Strain in Pulmonary Arterial Hypertension. The American Journal of Cardiology.

[7] Hayek Salim, Sims Daniel B., Markham David W., Butler Javed, Kalogeropoulos Andreas P. (2014). Assessment of Right Ventricular Function in Left Ventricular Assist Device Candidates. Circulation: Cardiovascular Imaging.

[8] Lisi Matteo, Cameli Matteo, Righini Francesca Maria, Malandrino Angela, Tacchini Damiana, Focardi Marta, Tsioulpas Charilaos, Bernazzali Sonia, Tanganelli Piero, Maccherini Massimo, Mondillo Sergio, Henein Michael Y. (2015). RV Longitudinal Deformation Correlates With Myocardial Fibrosis in Patients With End-Stage Heart Failure. JACC: Cardiovascular Imaging.

[9] Haddad François, Doyle Ramona, Murphy Daniel J., Hunt Sharon A. (2008). Right Ventricular Function in Cardiovascular Disease, Part II. Circulation.

[10] Murninkas Daniel, Alba Ana C., Delgado Diego, McDonald Michael, Billia Filio, Chan Wai S., Ross Heather J. (2014). Right Ventricular Function and Prognosis in Stable Heart Failure Patients. Journal of Cardiac Failure.

[11] Lang Roberto M., Badano Luigi P., Mor-Avi Victor, Afilalo Jonathan, Armstrong Anderson, Ernande Laura, Flachskampf Frank A., Foster Elyse, Goldstein Steven A., Kuznetsova Tatiana, Lancellotti Patrizio, Muraru Denisa, Picard Michael H., Rietzschel Ernst R., Rudski Lawrence, Spencer Kirk T., Tsang Wendy, Voigt Jens-Uwe (2015). Recommendations for Cardiac Chamber Quantification by Echocardiography in Adults: An Update from the American Society of Echocardiography and the European Association of Cardiovascular Imaging. Journal of the American Society of Echocardiography.

[12] BARENDSWAARD E C, PRPIC H, VAN DER WALL E E, CAMPS J A J, KEIZER H J, PAUWELS E K J (1991). Right Ventricle Wall Motion Abnormalities in Patients Treated with Chemotherapy. Clinical Nuclear Medicine.

[13] Grover Suchi, Leong Darryl P., Chakrabarty Adhiraj, Joerg Lucas, Kotasek Dusan, Cheong Kerry, Joshi Rohit, Joseph Majo X., DePasquale Carmine, Koczwara Bogda, Selvanayagam Joseph B. (2013). Left and right ventricular effects of anthracycline and trastuzumab chemotherapy: A prospective study using novel cardiac imaging and biochemical markers. International Journal of Cardiology.

[14] Tanindi A., Demirci U., Tacoy G., Buyukberber S., Alsancak Y., Coskun U., Yalcin R., Benekli M. (2011). Assessment of right ventricular functions during cancer chemotherapy. European Journal of Echocardiography.

[15] Ganame Javier, Claus Piet, Uyttebroeck Anne, Renard Marleen, D’hooge Jan, Bijnens Bart, Sutherland George R., Eyskens Bénédicte, Mertens Luc (2007). Myocardial Dysfunction Late After Low-Dose Anthracycline Treatment in Asymptomatic Pediatric Patients. Journal of the American Society of Echocardiography.

[16] Yağci-Küpeli Begül, Varan Ali, Yorgun Hikmet, Kaya Bariş, Büyükpamukçu Münevver (2012). Tissue Doppler and myocardial deformation imaging to detect myocardial dysfunction in pediatric cancer patients treated with high doses of anthracyclines. Asia-Pacific Journal of Clinical Oncology.

[17] Oliveira Guilherme H., Dupont Matthias, Naftel David, Myers Susan L., Yuan Ya, Tang W.H. Wilson, Gonzalez-Stawinski Gonzalo, Young James B., Taylor David O., Starling Randall C. (2014). Increased Need for Right Ventricular Support in Patients With Chemotherapy-Induced Cardiomyopathy Undergoing Mechanical Circulatory Support. Journal of the American College of Cardiology.

[18] Calleja Anna, Poulin Frédéric, Khorolsky Ciril, Shariat Masoud, Bedard Philippe L., Amir Eitan, Rakowski Harry, McDonald Michael, Delgado Diego, Thavendiranathan Paaladinesh (2015). Right Ventricular Dysfunction in Patients Experiencing Cardiotoxicity during Breast Cancer Therapy. Journal of Oncology.

[19] Boczar Kevin Emery, Aseyev Olexiy, Sulpher Jeffrey, Johnson Christopher, Burwash Ian G, Turek Michele, Dent Susan, Dwivedi Girish (2016). Right heart function deteriorates in breast cancer patients undergoing anthracycline-based chemotherapy. Echo Research and Practice.

[20] Abdar Esfahani Morteza, Mokarian Fariborz, Karimipanah Mohammad (2016). Alterations in the echocardiographic variables of the right ventricle in asymptomatic patients with breast cancer during anthracycline chemotherapy. Postgraduate Medical Journal.

[21] Chang WT, Shih JY, Feng YH, Chiang CY, Kuo YH, Chen WY, Wu HC, Cheng JT, Wang JJ, Chen ZC (2016). The Early Predictive Value of Right Ventricular Strain in Epirubicin-Induced Cardiotoxicity in Patients with Breast Cancer
.. Acta Cardiol Sin..

[22] Cardinale Daniela, Colombo Alessandro, Bacchiani Giulia, Tedeschi Ines, Meroni Carlo A., Veglia Fabrizio, Civelli Maurizio, Lamantia Giuseppina, Colombo Nicola, Curigliano Giuseppe, Fiorentini Cesare, Cipolla Carlo M. (2015). Early Detection of Anthracycline Cardiotoxicity and Improvement With Heart Failure Therapy. Circulation.

[23] Muraru Denisa, Onciul Sebastian, Peluso Diletta, Soriani Nicola, Cucchini Umberto, Aruta Patrizia, Romeo Gabriella, Cavalli Giacomo, Iliceto Sabino, Badano Luigi P. (2016). Sex- and Method-Specific Reference Values for Right Ventricular Strain by 2-Dimensional Speckle-Tracking Echocardiography. Circulation: Cardiovascular Imaging.

[24] Farsalinos Konstantinos E., Daraban Ana M., Ünlü Serkan, Thomas James D., Badano Luigi P., Voigt Jens-Uwe (2015). Head-to-Head Comparison of Global Longitudinal Strain Measurements among Nine Different Vendors. Journal of the American Society of Echocardiography.

